# Presentation of Neuro-COVID is Broad and Pathogenesis Diverse

**DOI:** 10.5811/westjem.2021.1.50893

**Published:** 2021-04-08

**Authors:** Josef Finsterer

**Affiliations:** Klinik Landstrasse, Messerli Institute, Department of Neurology, Vienna, Austria

To the Editor:

With interest we read the review article by Valiuddin et al. about the neurological implications of coronavirus disease 2019 (COVID-19) (neuro-COVID).^1^ The authors listed ischemic stroke, transverse myelitis, seizures, acute hemorrhaghic necrotising encephalopathy (AHNE), acute disseminated encephalo-myelitis (ADEM), posterior reversible encephalopathy syndrome (PRES), myasthenia, and sinus venous thrombosis as central nervous system (CNS) manifestations, and hyposmia/hypogeusia, Guillain-Barré syndrome, facial palsy, ophthalmoparesis, and neuropathy, as peripheral nervous system (PNS) manifestations of COVID-19.^1^ We have the following comments and concerns.

The review is comprehensive but does not include the entire spectrum of neuro-COVID-19. Several neurological manifestations of COVID-19 in the CNS and PNS were not discussed. CNS disorders not included in the review were intracerebral bleeding,^2^ cerebral vasculitis,^3^ acute cerebral demyelination,^4^ headache,^5^ myoclonus-ataxia syndrome,^6^ limbic encephalitis,^7^ cytokine release syndrome,^8^ delirium,^9^ and psychosis.^10^ Peripheral nervous system disorders not included in the review were isolated oculomotor, trochlear, facial, or hypoglossal nerve palsy,^11^ myositis/dermatomyositis,^12^ myopathy,^13^ and rhabdomyolysis.^14^

There was no discussion about the putative delineation between neurological disorders due to direct attack of the virus (primary manifestations), secondary CNS/PNS disorders due to the immune response (secondary manifestations), and those occurring as a side effect of the treatment or involvement of other organs than the CNS (tertiary manifestations). Whether such a distinction is truly permissible is under debate. Limited data from animal and basic science research are currently available. Disregarding this debate, there are indications that the virus enters the CNS via the blood brain barrier (BBB) or via retrograde invasion along peripheral nerves, as mentioned in the review.^1^,^15^ There are even indications that the virus disrupts the BBB.^16^ Interestingly, in most of the CNS disorders claimed to have been triggered by direct contact of the virus with CNS structures, investigations of the cerebro-spinal fluid (CSF) did not confirm the presence of the virus in the CSF. Absence of the virus in the CSF has been explained by rapid entering of the virus intra-cellularly, thus being present in the CSF only temporarily for a short time. Accordingly, virus-RNA has been found inside neurons and glial cells.^17^

Since the infection triggers an immense immune reaction, it has been speculated that the immune reaction is responsible for many or most of the neurological comorbidities in COVID-19 patients. This is even the case for encephalitis, which is often characterised by a negative polymerase chain reaction test for the virus in the CSF and thus interpreted as immune encephalitis. Other immune-mediated CNS/PNS disorders in COVID-19 include limbic encephalitis, cerebral vasculitis, AHNE, cytokine-release syndrome, myoclonus-ataxia syndrome, ADEM, delirium, psychosis, transverse myelitis, isolated cranial nerve palsy, myositis, or myasthenia.

Side effects of treatment have to be clearly delineated from primary or secondary neurological manifestations of the viral infection. Adverse reactions to treatment particularly manifest in the PNS. CNS/PNS diseases due to adverse reactions include cerebral hypoxia, critical ill neuropathy, critical ill myopathy, myasthenic syndrome, myopathy, and toxic neuropathy. Agents with a neurotoxic potential include hydro-chloroquine, which may trigger toxic myopathy, steroids, which may induce mitochondrial myopathy, tocilizumab, which may trigger myositis, and azithromycin, which may cause rhabdomyolysis.

Though the authors state that myasthenia gravis (MG) can be a complication of COVID-19, they cite the paper by Anand et al. who only described five patients with pre-existing MG (four due to antibodies against the acetyl-choline receptor, and one due to antibodies against tyrosine-kinase) who experienced an infection with severe acute respiratory syndrome coronavirus 2 (SARS-CoV-2). Thus, this article cannot be taken as an example of SARS-CoV-2 triggered MG. More appropriate for documenting MG triggered by SARS-CoV-2 is the study by Restivo et al. who described three previously neurologically normal patients who developed confirmed MG after onset of the classical manifestations of COVID-19.^18^

Finally, we do not agree with the listing of MG among the CNS disorders triggered by SARS-CoV-2 in Table 1.^1^ Myasthenia gravis is a prototypic PNS disorder and should not be mentioned among the CNS but the PNS disorders.

Overall, the interesting review has a number of limitations, which need to be accomplished before drawing final conclusions. The spectrum of neurological disease triggered by SARS-CoV-2 is broader than anticipated. Neurological disease possibly results from a direct virus attack, from the immune response, or from side effects of the treatment of pulmonary manifestations.
